# Real-time changes in rib cage expansion and use of abdominal mechanical stimulation in newborns: a quasi-experimental study

**DOI:** 10.1590/1984-0462/2024/42/2023032

**Published:** 2023-12-22

**Authors:** Jaiana Xavier Santos, Pedro Ykaro Fialho Silva, Maria Clara Lima da Cruz, Bianca Fernandes Vasconcelos e Silva, Ingrid Guerra Azevedo, Silvana Alves Pereira

**Affiliations:** a Universidade Federal do Rio Grande do Norte, Natal, RN, Brazil.; b Universidad Católica de Temuco, Temuco, Araucanía, Chile.

**Keywords:** Infant, newborn, Respiratory mechanics, Pulmonary ventilation, Recém-nascido, Mecânica respiratória, Ventilação pulmonar

## Abstract

**Objective:**

To assess the rib cage expansion and respiratory rate in newborns using an abdominal stabilization band.

**Methods:**

The study included 32 newborns of both genders, with gestational age between 35 and 41 weeks. The abdominal stabilization band was used for 15 minutes between the xiphoid process and the anterosuperior iliac crest, with an abdominal contention 0.5cm smaller than the abdominal circumference. The rib cage expansion was evaluated by a breathing transducer (Pneumotrace II™) three minutes before using the band, during the use (15 minutes), and ten minutes after removing the band. The Shapiro-Wilk test verified data normality, and the Wilcoxon test compared the variables considering rib cage expansion and respiratory rate. Significance was set to p<0.05.

**Results:**

There was an increase in respiratory rate when comparing before and ten minutes after removing (p=0.008) the abdominal stabilization band, as well as when comparing during its use and ten minutes after its removal (p=0.001). There was also an increase in rib cage expansion when comparing before and during the use of the abdominal stabilization band (p=0.005).

**Conclusions:**

The use of the abdominal stabilization band promoted an increase in the rib cage expansion and respiratory rate in the assessed newborns and may be a viable option to improve the respiratory kinematics of this population.

## RESUMO

**Objetivo:**

Avaliar a expansibilidade torácica e a frequência respiratória em recém-nascidos que fizeram uso de uma faixa de estabilização abdominal.

**Métodos:**

O estudo incluiu 32 recém-nascidos de ambos os sexos, com idade gestacional entre 35 e 41 semanas. A faixa de estabilização abdominal foi mantida por 15 minutos entre o processo xifoide e a espinha ilíaca anterossuperior, com contenção abdominal 0,5 cm menor do que a circunferência abdominal. A expansibilidade torácica foi avaliada por um transdutor piezoelétrico (Pneumotrace II™) 3 minutos antes do uso da faixa, durante 15 minutos, e 10 minutos após sua retirada. A normalidade das variáveis foi testada pelo teste de Shapiro-Wilk e a análise comparativa da expansibilidade torácica e da frequência respiratória foi realizada por meio do teste *t* pareado, considerando-se p<0,05.

**Resultados:**

Houve aumento da frequência respiratória quando comparados os tempos antes do uso da faixa e 10 minutos após a retirada (p=0,008), bem como quando comparados os tempos durante o uso e 10 minutos após a retirada da faixa (p=0,001). Houve aumento da expansibilidade torácica quando comparados os tempos antes e durante o uso da faixa (p=0,005).

**Conclusões:**

O uso da faixa de estabilização abdominal conferiu aumento da expansibilidade torácica e da frequência respiratória nos recém-nascidos estudados, podendo ser uma opção viável para a melhora da cinemática respiratória dessa população.

## INTRODUCTION

The biomechanical immaturity and neural control of the respiratory system of newborns (NB) put them in a vulnerable position during situations of increased ventilatory demand.^1^ The horizontal position of the costal margins decreases the diaphragmatic zone of apposition and the efficiency of the intercostal muscles, and unlike the lung, which is less compliant, less mineralized ribs imply a more compliant and deformable rib cage.^2^ All of these characteristics offer less stability to the chest wall and predispose to the emergence of paradoxical chest wall movements, which increase the diaphragmatic work.^3^

Unlike in adults, rib cage expansion in the neonatal period obtains advantages from the rib cage response.^4^ Since NB’ ribs are horizontal, which alters the length-tension relationship of the muscles attached to the rib cage.^3,5^ Conditions that offer greater support for the rib cage can improve the force of contraction and increase thoracic expansion.^4^ Lanza et al.^6^ demonstrated that chest wall mobility in healthy individuals is related to respiratory muscle strength and lung function. However, the authors studied only adult individuals.

Some studies have tried to demonstrate that rib cage stabilization in NB can positively contribute to cardiorespiratory indications,^4,7,8^ promoting an increase in the response to oxygen saturation or a decrease in heart^4^ and respiratory rates,^7^ as well as thoracoabdominal asynchrony.^8^ However, little is known about the behavior of rib cage expansion after stabilization.

In the last years, significant attention has been directed to methods for non-invasive measurement of the chest wall movements and patterns.^9,10^ The use of respiratory transducers is a promising method to detect changes in chest expansion and an important variable for thoracoabdominal kinematics.^11^

Thus, this study hypothesized that the use of an abdominal band can stabilize the NB’ rib cage. We believe that restricting the movement of the floating ribs during spontaneous breathing can modulate respiratory behavior, supporting the thoracoabdominal synchrony and increasing rib cage expansion. Furthermore, we believe that the respiratory transducer may be an effective method to assess this outcome. In this context, the aim of this study was to assess rib cage expansion and respiratory rate in newborns using an abdominal stabilization band.

## METHOD

This quasi-experimental study was conducted in the neonatal units of a Maternity School between July and December 2020. Parents were informed about the purposes, benefits, and risks before enrollment, and all agreed to participate and signed a consent form. The study was approved by a Research Ethics Committee (protocol number 3.302.184) in accordance with the Declaration of Helsinki.

The study included 32 NB, of both genders, with gestational age between 35 and 41 weeks, without ventilatory support or supplemental oxygen at the time of evaluation. Those with chest wall deformities or skin lesions, necrotizing enterocolitis, hyperthermia, hypothermia, abdominal diseases, congenital diaphragmatic hernia, or presenting a chest drain tube were excluded. Those who missed the study procedures due to intense inconsolable crying were also excluded from the analysis.

Data collection was performed in an adapted portable crib with the NB in the supine position. The rib cage perimeter was measured before placing the stabilization band using a non-extensible measuring tape (in centimeters), with the xiphoid process as a reference point.^12^

**Figure 1:**
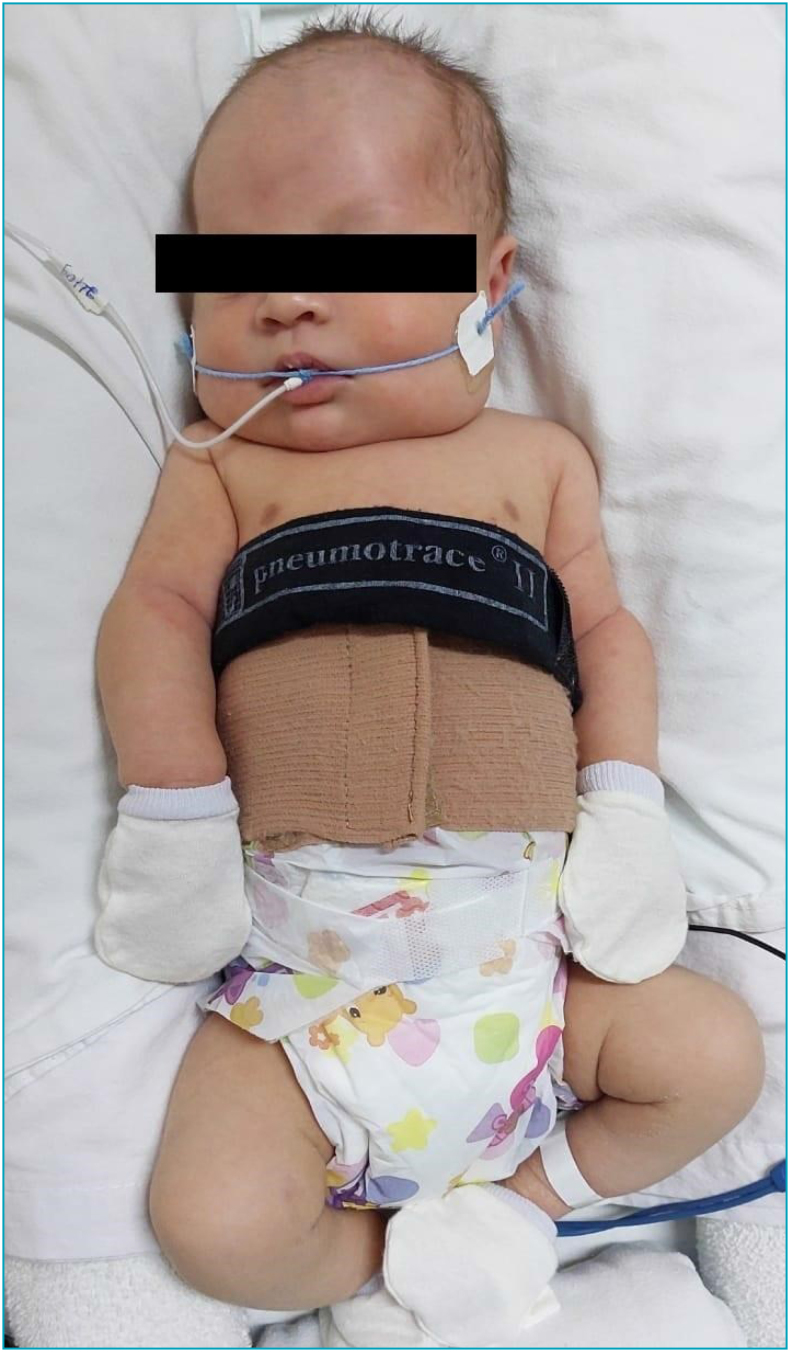
Newborn using the abdominal stabilization band (beige color) and Pneumotrace II™ (black color) to assess the rib cage expansion.

For rib cage stabilization, a hypoallergenic abdominal band (cotton, polyamide, and elastodiene), with velcro closure, and marked in centimeters in the horizontal direction (Figure 1) was used. At the end of the inspiratory phase, the band was adjusted between the xiphoid process and the anterosuperior iliac crest, with an abdominal contention 0.5cm smaller than the abdominal circumference measured without the band. The measure of abdominal circumference was obtained with a non stretchable tape wrapped around the xiphoid process level while NB were in a supine position.

The abdominal stabilization band was maintained for 15 minutes, and the rib cage expansion (mV) and respiratory rate (per minute) were evaluated using a respiratory transducer (Pneumotrace II™, UFI, Morro Bay, CA, United States) attached to the abdominal stabilization band and with amplification and filtering acquired by Powerlab^®^ (ADInstruments, Australia). The respiratory belt transducer is designed to measure changes in chest diameter and produces a linear voltage proportional to changes in length resulting from breathing.

**Figure 2:**
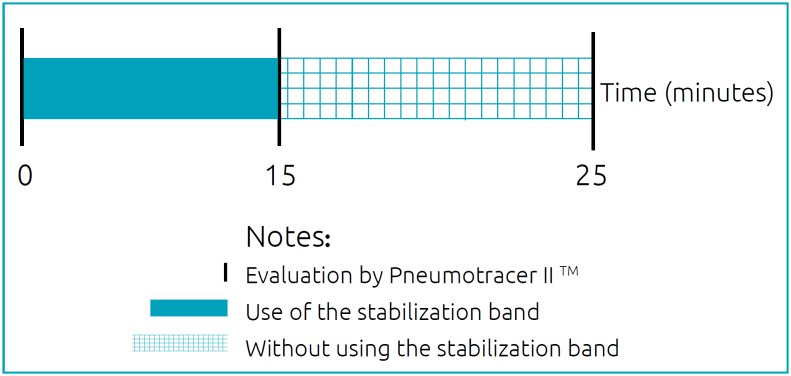
Study procedures.

The respiratory transducer was positioned on the thoracic area (xiphoid process, in the intermammary cleft), and chest expansion was measured three minutes before the band use, during its use (after 15 minutes of use) and ten minutes after its removal (Figure 2), using a sampling frequency of 20 millivolts. A blinded researcher measured offline the results obtained by the Pneumotrace II (respiratory rate and rib cage expansion) at the three different moments assessed in this study.

Statistical analysis was performed using the Statistical Package for the Social Sciences software (SPSS Inc., Chicago, IL, USA), version 20.0. Data normality was tested using the Shapiro-Wilk test, showing a not normal distribution, and data were presented as median and interquartile range. The Wilcoxon test compared the variables considering rib cage expansion and respiratory rate at three different moments, independently: before using the abdominal band, immediately after the abdominal band, and ten minutes after the last measurement. For all statistical analyses, a significance level of <0.05 was adopted. A post-hoc analysis considering a Cohen’s d effect size of 0.95 between rib cage expansion before and ten minutes after the abdominal stabilization band and α error of 0.02 showed a statistical power of 53% for this study.

## RESULTS

During the period of this study, a total of 90 NB were hospitalized and 35 met the inclusion criteria. Three NB were excluded from the study due to intense inconsolable crying. Out of the 32 NB who completed the protocol, 14 were female, and 21 were born by cesarian section. Table 1 presents the sample description.

**Table 1:** Sample characteristics (n=32).

Characteristics	Mean±SD	Median	Minimum	Maximum
Days of life	2.5±1.27	3	1	5
Gestational age at assessment (weeks)	37.3±1.73	37	35	40
Weight at day of assessment (g)	3059±650	3020	1930	4290
1^st^ minute Apgar score	7±1.41	8	4	9
5^th^ minute Apgar score	8±0.60	9	7	10
Chest circumference (cm)	32.4±2.4	32.5	28	36

SD: standard deviation.

No differences in respiratory rate were observed before and during the use of the abdominal stabilization band. However, there was a significant increase in respiratory rate when comparing before and ten minutes after removing (p=0.008) the abdominal stabilization band, as well as when comparing during its use and ten minutes after its removal (p=0.001).

When comparing rib cage expansion before and during the use of the abdominal stabilization band, a significant increase was observed (p=0.005). However, although there was also an increase in rib cage expansion when comparing before and ten minutes after using the abdominal stabilization band, this difference did not reach statistical significance. Values from the rib cage expansion and respiratory rate before, during and ten minutes after using the abdominal stabilization band, as well as the Wilcoxon test, are shown in Table 2.

**Table 2:** Rib cage expansion and respiratory rate before, during, and ten minutes after using the abdominal stabilization band (n=32).

	Time	Median	IQ25–75	p-value
Rib cage expansion (mV)	Before	4.26	3.63–4.72	0.005
	During ASB	4.46	4.10–4.93	
	Before	4.26	3.63–4.72	0.100
	Ten minutes after the ASB	4.40	4.03–5.44	
	During ASB	4.46	4.10–4.93	0.240
	Ten minutes after the ASB	4.40	4.03–5.44	
Respiratory rate	Before	35.50	31.25–37.65	0.594
	During ASB	34.38	32.00–37.85	
	Before	35.50	31.25–37.65	0.008
	Ten minutes after the ASB	44.00	34.52–53.47	
	During ASB	34.38	32.00–37.85	0.001
	Ten minutes after the ASB	44.00	34.52–53.47	

IQ: interquartile range; ASB: abdominal stabilization band; mV: millivolts

## DISCUSSION

The present study aimed to assess, in a short term, the response of rib cage expansion in NB during and after using an abdominal stabilization band. The results demonstrate that there was a significant increase in respiratory rate when comparing before and ten minutes after removing the abdominal stabilization band, as well as when comparing during its use and ten minutes after its removal. Rib cage expansion increased significantly while the band was being used. These findings can be attributed to the activation of chemoreceptors and baroreceptors, as well as the biomechanical effects induced by the utilization of the abdominal stabilization band.^13,14^

The changes that continually occur in ventilation are the result of the integration of afferent and efferent signals between chemoreceptors, baroreceptors, upper airways and lungs, which have influence in the control of breathing through the modulation of the neuronal networks that make up the respiratory center, a structure located in the brainstem.^15^ However, during the neonatal period, this communication between the respiratory center and lungs is still in an immature state, which could potentially explain the delayed response (observed after 10 minutes) to mechanical stimulation.^13^

Moreover, the increase in the respiratory rate could also be explained by the mechanical adjustment provided by the band, which restricts the abdominal wall, limiting the diaphragmatic excursion and leading to a compensatory activation of the respiratory center.^16^

Despite the observed increase in respiratory rate, no increase in respiratory distress was observed in the assessed infants. In the short term, this enhanced respiratory rate, along with improved stabilization of the thoracoabdominal compartment, may contribute to increased rib cage expansion. This finding is particularly noteworthy, as it aligns with the observed increase in rib cage expansion during the use of the abdominal stabilization band.

Neural control plasticity can favor interventions that minimize the mechanical disadvantages experienced by neonates, especially in premature newborns, who present worse thoracoabdominal synchrony response in situations of increased inspiratory resistive load.^17^ Considering the anatomical conditions of the respiratory mechanics in preterm NB, such as the horizontal position of the costal margins, less mineralized ribs and a more compliant and deformable rib cage,^18,19^ which are not favorable for the maintenance of a stable lung volume, the abdominal stabilization band utilized in this study, could support the respiratory physiotherapy sessions.

Manual techniques or devices that promote improvement in oxygenation and ventilation levels have a physiological rationale highlighted by the action of baroreceptors.^20,21^ Studies suggest that NB are more sensitive to peripheral stimuli on the regulation of neural respiratory control and that baroreceptors may be functional even in the neonatal period, but they go through a process of structure sophistication over time.^14,22^

Other studies corroborate with our hypothesis and have shown that mechanical adjustments performed by physiotherapists^7^ or adapted accessories^4,23^ for stabilization of the costal margins may improve some respiratory parameters, such as oxygen saturation,^4^ thoracoabdominal asynchrony,^8^ respiratory rate, and respiratory distress.^7^ Therefore, we hypothesize that concomitantly with the use of the abdominal stabilization band, the physiotherapist’s hands could be employed to enhance another aspect of respiratory function,^19^ potentially improving respiratory outcomes within a single session. However, the currently existing literature lacks sufficient data regarding the impact of stabilization on rib cage expansion in newborns.

Associated with this mechanical condition, the development of respiratory control comprises complex and dynamic interactions^13^ and may be subject to structural or functional changes during critical maturation periods.^5^ Critical periods represent a moment of greater flexibility in response to respiratory control, and it is supposed that at this moment, in which there is a greater susceptibility to alterations, there is also a greater capacity for reversibility (*e.g*., increased variability in oxygen levels, which are inherent to neonatal intensive care).^24^

Although different hypotheses were presented here to justify the variability in rib cage expansion and respiratory rate in the evaluated NB, we recognize that this study has some limitations, such as the fact that the protocol start order was not randomized. In addition, the absence of a thoracic belt for a more comprehensive assessment of the thoracoabdominal synchrony is also a limitation that could be explored in future studies. On the other hand, this was the first study assessing the rib cage expansion in NB using an abdominal stabilization band, a low-cost intervention which may help in understanding the responses of respiratory biomechanics in this population.

It is important to highlight that the increase in rib cage expansion was not sustained after the removal of the abdominal stabilization band, which leads to the need to investigate the lasting effects of the manual and instrumental techniques used in respiratory physiotherapy. Besides, studying chest maneuvers to help professionals to optimize health care in a neonatal care is crucial,^19^ since the literature is still uncertain about respiratory physiotherapy techniques which can minimize the impacts of mechanical ventilation in NB.^25–27^ Therefore, introducing the abdominal stabilization band, as an alternative procedure, could be a start for new clinical trials with preterm NB or NB with complex respiratory conditions, such as respiratory distress syndrome and bronchopulmonary dysplasia.

Based on this, it was possible to observe that the use of the mechanical stimulus with the abdominal stabilization band in the studied NB increased rib cage expansion during its use, as well as increased the respiratory rate during and after the use of the band.
